# Beyond the pump: a narrative review of the acute molecular, metabolic, and neuropsychological responses to a bout of resistance exercise

**DOI:** 10.7717/peerj.21827

**Published:** 2026-10-16

**Authors:** James P. Fisher

**Affiliations:** 1 The Exercise Coach USA, Smithville, TN, United States of America; 2 School of Allied Health and Exercise Sciences, Bournemouth, Poole, Dorset, United Kingdom

**Keywords:** Myokine, Resistance exercise, Resistance training, Strength training, Systemic health

## Abstract

**Background:**

Resistance training is widely recognized for its chronic adaptations, including strength, muscle hypertrophy, and improvements in musculoskeletal health. However, a single bout of resistance exercise also elicits a range of acute physiological and psychological responses across multiple systems. These immediate responses have generally been examined within individual physiological domains, with less attention given to their coexistence and temporal overlap following a single session.

**Methods:**

This narrative review synthesizes evidence relating to the acute systemic effects of a single bout of resistance exercise. Literature was identified through iterative searches of electronic databases and reference lists, with emphasis on primary human studies directly examining acute responses, alongside relevant systematic reviews, meta-analyses, and mechanistic literature.

**Results:**

Acute resistance exercise induces responses across endocrine and myokine, metabolic, vascular, pain-related, psychological, cognitive, and sleep-related domains. Contracting skeletal muscle releases myokines and other signalling molecules with potential systemic actions. Post-exercise energy expenditure and muscle protein synthesis may remain elevated during recovery, while glucose uptake and insulin sensitivity are enhanced. Acute resistance exercise can also produce exercise-induced hypoalgesia, reduce state anxiety, improve aspects of executive function and cognitive performance, and may influence sleep continuity and neurophysiological activity. These responses differ in their evidential basis and physiological significance, encompassing directly observed short-term effects, proposed regulatory mechanisms, and more tentative hypotheses regarding longer-term implications.

**Conclusion:**

A single bout of resistance exercise produces temporally overlapping responses across multiple physiological and psychological systems. These responses should not be assumed to represent adaptive remodelling or direct mechanisms of chronic health benefit. Nevertheless, integrating them provides a broader perspective on the immediate multisystem consequences of resistance exercise and may help inform future research examining how repeated acute responses relate to longer-term adaptation and health outcomes.

## Introduction

Resistance training (RT) has traditionally been conceptualized as a stimulus for long-term structural adaptations to strength, muscle hypertrophy, and bone mineral density (McLeod et al., 2024; O’Bryan et al., 2022). However, over recent decades an expanding body of epidemiological and clinical research has demonstrated that the chronic benefits of RT extend well beyond musculoskeletal remodelling (Westcott, 2012). Regular participation in resistance exercise is associated with improved glycaemic control and insulin sensitivity, reductions in visceral adiposity, enhanced vascular function, preservation of lean mass with aging, and improvements in mental health and cognitive performance (Fisher et al., 2017). In combination, resistance training is associated with reductions in all-cause, cardiovascular, and cancer mortality (Shailendra et al., 2022; Shailendra et al., 2024; Momma et al., 2022). The physiological adaptations position RT not merely as a modality for athletic performance or aesthetic change, but as a cornerstone intervention in preventive and rehabilitative medicine.

The prevailing explanatory framework for these health benefits centres on cumulative structural adaptation over weeks to months of repeated training. Chronic improvements in muscle mass increase resting metabolic rate and glucose disposal capacity (McPherron et al., 2013); enhanced neuromuscular strength improves functional independence (Kirk, Steele & Fisher, 2024); and long-term anti-inflammatory and vascular adaptations reduce cardiometabolic risk (Lopez-Jaramillo et al., 2023). Yet this emphasis on chronic remodelling may inadvertently obscure a more immediate and equally important dimension of resistance exercise physiology: the acute systemic responses elicited by a single bout of muscular contraction.

Although previous reviews have examined selected acute responses to resistance exercise, these have generally focused on specific systems or outcomes (Cavarretta, Hall & Bixby, 2019; Wilke et al., 2019; Haunhorst et al., 2022). Other reviews have examined specific resistance exercise modalities or compared acute responses under different training conditions (Meirelles & Gomes, 2004; De Queiros et al., 2023). To our knowledge, no previous review has sought to synthesize the acute molecular, metabolic, vascular, immunological, neuropsychological, and sleep-related responses to a single bout of conventional resistance exercise within a single broad, multisystem framework. The present review therefore takes a deliberately integrative narrative approach, with the objective of considering how these temporally overlapping responses may collectively contribute to the broader physiological significance of an individual resistance exercise session.

Emerging evidence indicates that even one session of resistance exercise can elicit acute responses across physiological and psychological domains. For example, contracting skeletal muscle releases myokines with systemic signalling properties (Zunner et al., 2022); post-exercise energy expenditure remains elevated (Schuenke, Mikat & McBride, 2002); glucose uptake and insulin sensitivity improve (Brown et al., 2020); muscle protein turnover accelerates (Phillips, 2014); vascular tone transiently adjusts (Francois et al., 2016); pain perception is reduced (Focht & Koltyn, 2009); and mood and cognitive performance are enhanced (Amore et al., 2025; Basso & Suzuki, 2016).

Acute physiological responses should not, however, be interpreted as synonymous with adaptive remodelling. A single bout of resistance exercise imposes mechanical, metabolic, haemodynamic, and neuroendocrine stress, eliciting a range of transient perturbations and compensatory responses across multiple systems. Some of these responses may have immediate functional or psychological consequences, whereas others may primarily reflect the restoration of homeostasis or provide signals that could potentially contribute to longer-term adaptation when exercise is repeated. The relationship between any individual acute response and subsequent chronic adaptation, however, cannot be assumed.

Accordingly, this review examines the acute systemic effects of a single bout of resistance exercise across molecular, metabolic, vascular, immunological, pain-related, psychological, cognitive, and sleep-related domains. The aim is to synthesize these temporally overlapping responses within a multisystem framework, distinguishing where possible between directly observed acute effects, proposed regulatory mechanisms, and hypotheses regarding their potential relevance to longer-term adaptation and health. The principal physiological domains and acute responses considered are summarised in Table 1 and integrated within the multisystem conceptual framework presented in Fig. 1.

**Table 1:** Summary of acute multisystem responses to a single bout of resistance exercise, the strength of supporting evidence, and key knowledge gaps.

**Domain**	**Principal acute responses discussed in this review**	**Representative references**
Endocrine and myokine responses	Acute contraction induces the release of myokines and other signalling molecules with autocrine, paracrine, and endocrine actions, including potential metabolic, anti-inflammatory, and immune-related effects	Zunner et al. (2022); Mănescu et al. (2026); Ringleb et al. (2024); Pedersen & Febbraio (2008); Shen et al. (2022); Ghanemi, Yoshioka & St-Amand (2023); Becknell & Caligiuri (2005)
Vascular	A single bout of resistance exercise produces transient haemodynamic and vascular responses, including a short-lived increase in arterial stiffness followed by a reduction in resting blood pressure during recovery	Jurik, Zebrowska & Stastny (2021); Aly & Yeung (2023); Rezk et al. (2006); MacDonald (2002); Chen et al. (2009)
Metabolic	A single bout of resistance exercise acutely increases muscle protein synthesis and energy expenditure while enhancing glucose uptake and insulin sensitivity, with additional changes in osteocalcin and appetite-related signalling during recovery.	Schuenke, Mikat & McBride (2002); Brown et al. (2020); Phillips (2014); Phillips et al. (1997); Atherton & Smith (2012); Bodine et al. (2001); Bond (2016); Richter & Hargreaves (2013); Jensen et al. (2011); Lee et al. (2007); Scott et al. (2011); Wei et al. (2014); Mohammad Rahimi et al. (2021); Thomas et al. (2024); Broom et al. (2009); Balaguera-Cortes et al. (2011); Schubert et al. (2014); Panissa et al. (2024)
Pain	A single bout of resistance exercise can acutely reduce pain perception, increasing pain thresholds and tolerance through exercise-induced hypoalgesia involving endogenous pain-modulatory mechanisms.	O’Bryan et al. (2022); Wewege & Jones (2021); Baiamonte et al. (2017); Koltyn et al. (2014)
Psychological Wellbeing	A single bout of resistance exercise can acutely reduce state anxiety and modestly improve affect, with evidence also suggesting short-term changes in mood and emotional wellbeing.	Amore et al. (2025); Strickl & Smith (2014); Cavarretta, Hall & Bixby (2019)
Cognitive Enhancement and Executive Function	A single bout of resistance exercise can acutely enhance executive function and global cognitive performance, with the most consistent effects reported for inhibitory control and cognitive flexibility.	Wilke et al. (2019); Soga et al. (2018); Engeroff, Banzer & Niederer (2022); Anders et al. (2021)
Sleep	A single bout of resistance exercise may improve sleep continuity, including faster sleep onset and fewer nocturnal awakenings, while also producing modest changes in sleep architecture and electroencephalogram (EEG) activity.	Alley et al. (2015); Perrier et al. (2024)

**Notes.**

As this was a broad narrative review rather than an exhaustive systematic assessment, no formal study selection, risk-of-bias assessment, or certainty-of-evidence evaluation was undertaken. References are provided as representative sources supporting the principal findings within each domain and are not intended to be exhaustive.

## Methods

This article was conducted as a narrative review aimed at synthesizing evidence relevant to the acute systemic effects of a single bout of resistance exercise. Given the mechanistic and translational focus of the manuscript, a narrative approach was selected to allow integration of molecular, physiological, metabolic, and psychological findings across diverse methodological paradigms, including randomized controlled trials, crossover designs, mechanistic laboratory studies, and systematic reviews.

Relevant literature was identified through searches of electronic databases including PubMed, Scopus, Google Scholar, and Web of Science. Searches were conducted using combinations of the following keywords: acute, single bout, resistance exercise, resistance training, myokines, post-exercise oxygen consumption, glycaemic response, insulin sensitivity, muscle protein synthesis, metabolomics, arterial stiffness, pulse wave velocity, exercise-induced hypoalgesia, acute anxiety, mood response, cognitive performance, executive function, and sleep. The literature search considered studies published prior to January 2026.

**Figure 1:**
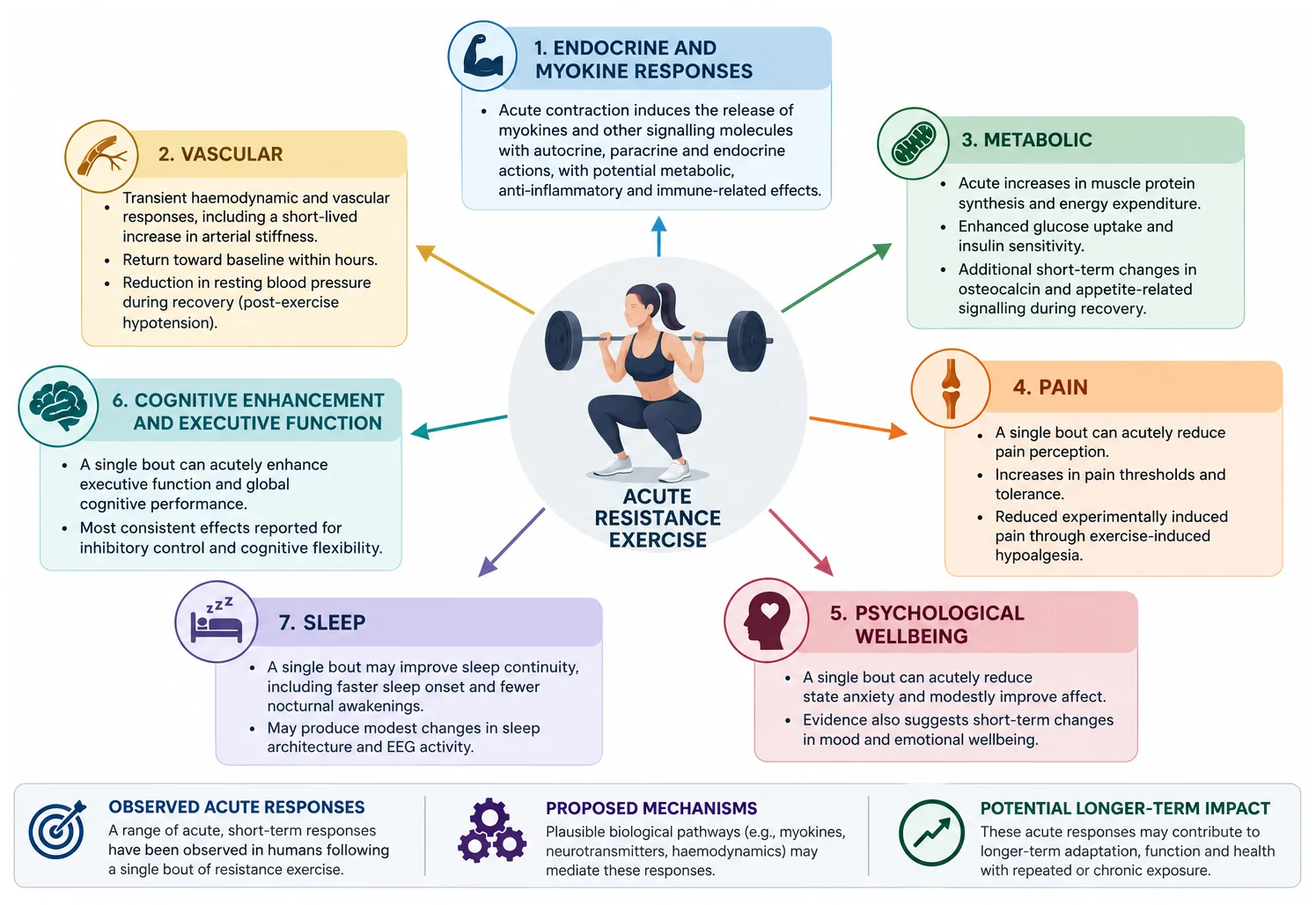
Conceptual framework of the acute multisystem responses to a single bout of resistance exercise.

Study identification and selection were conducted iteratively in keeping with the narrative review design. Initial searches were undertaken to identify relevant systematic reviews, major narrative reviews, and key primary studies addressing the acute effects of a single bout of resistance exercise within each physiological domain. Primary studies were then prioritised where they provided direct evidence of acute responses to conventional resistance exercise in human participants. Where available, systematic reviews and meta-analyses were used to provide broader synthesis of the literature and to identify additional relevant primary studies.

Reference lists of key articles were subsequently screened, and citation chaining was used to identify further studies of relevance. Studies were prioritised according to their direct relevance to the objective of the review, methodological design, measurement of acute outcomes, and relevance to the proposed multisystem framework. Greater emphasis was placed on controlled experimental studies, randomized crossover designs, and studies using validated physiological, biochemical, cognitive, or psychological outcome measures. Foundational or mechanistic studies were also included where necessary to provide context for the interpretation of acute findings.

The literature considered for inclusion focused primarily on evidence addressing acute responses to a single resistance training session or resistance exercise bout, including physiological, metabolic, vascular, endocrine, immune, psychological, cognitive, and sleep-related outcomes in human participants. Primary studies directly examining these acute responses formed the principal evidential basis of the review. Systematic reviews, meta-analyses, and major narrative reviews were additionally considered where relevant to provide broader synthesis, contextual background, or to identify further primary literature. Animal studies and chronic training interventions without acute outcome reporting were not a primary focus but were considered where they provided relevant mechanistic context.

Given the narrative review design, a formal risk-of-bias assessment and quantitative meta-analysis were not performed. The review was not intended as an exhaustive systematic assessment of all available evidence within each physiological domain. Accordingly, direct findings from systematic reviews, meta-analyses, and controlled experimental studies were distinguished from mechanistic and hypothesis-generating evidence used to provide biological context. Rather, relevant literature was selected and synthesised to address the objective of the review, with greater emphasis placed on studies employing controlled experimental designs and validated outcome measures where appropriate to the outcome being considered. Findings were synthesised thematically across systems to develop an integrated conceptual framework describing the multisystem acute responses to resistance exercise.

The acute response to resistance exercise spans multiple physiological domains and measurement scales. A narrative synthesis was therefore used to facilitate interdisciplinary and mechanistic integration across these domains. This approach was deemed appropriate since the objective was conceptual synthesis and integration rather than exhaustive identification of all available evidence, formal evidence grading, or effect size quantification.

## Endocrine and Myokine Responses

Skeletal muscle is increasingly recognized as an endocrine organ (Zunner et al., 2022). During and immediately following contraction, muscle fibers release signalling molecules collectively termed myokines. These molecules exert autocrine, paracrine, and endocrine effects, influencing distant tissues including adipose tissue, liver, vasculature, immune cells, and the central nervous system (Zunner et al., 2022). However, the interpretation of exercise-responsive circulating factors requires caution, as changes in circulating concentrations do not necessarily establish skeletal muscle as the sole source of a signal or demonstrate a direct effect in a target tissue. The timing, tissue origin, and biological context of individual responses may therefore influence their physiological interpretation (Mănescu et al., 2026).

The expanding catalogue of myokines and other exercise-responsive signalling molecules, and their involvement in anti-inflammatory, metabolic, and anticancer processes have been described in detail (Mănescu et al., 2026; Ringleb et al., 2024). The following examples are therefore intended to illustrate breadth of potential systemic signalling rather than provide an exhaustive catalogue of exercise-responsive mediators. Interleukin-6 (IL-6), when released acutely during contraction, exerts anti-inflammatory effects, stimulates glucose uptake and lipid oxidation while suppressing pro-inflammatory cytokines (Pedersen & Febbraio, 2008). Irisin promotes metabolic flexibility and adipose tissue browning (Shen et al., 2022). Secreted protein acidic and rich in cysteine (SPARC) has demonstrated anti-tumour properties, potentially through apoptosis, suggesting possible role for contraction-related signalling in processes relevant to oncological risk (Ghanemi, Yoshioka & St-Amand, 2023). Finally, IL-15 and IL-21 further play a role in immune surveillance and tumour clearance through the activation and proliferation of natural killer (NK) cells (Becknell & Caligiuri, 2005).

Importantly, these signalling cascades occur during and immediately after a single session. Thus, acute resistance exercise initiates systemic communication between skeletal muscle and peripheral organs, with these transient signalling responses potentially influencing metabolic, inflammatory, and other cellular processes.

## Vascular Responses

While resistance exercise elicits numerous favourable acute metabolic and neuroendocrine responses, its vascular effects require nuanced interpretation. While engagement in RT can serve to reduce blood pressure (MacDonald et al., 2016) and improve endothelial function (Silva et al., 2021), there has historically been concern regarding a brief transient increase in arterial stiffness, commonly assessed *via* pulse wave velocity (PWV). A systematic review and meta-analysis by Jurik, Zebrowska & Stastny (2021) provides comprehensive insight into both acute and chronic vascular responses to resistance training. A single bout of resistance exercise consistently produced a transient increase in carotid–femoral pulse wave velocity (cfPWV), although, in most cases, PWV values returned to baseline within 20–60 min post-exercise (Jurik, Zebrowska & Stastny, 2021).

However, these transient increases likely reflect functional rather than structural vascular responses (Jurik, Zebrowska & Stastny, 2021; Aly & Yeung, 2023). Resistance exercise can acutely elevate systolic blood pressure, cardiac output, intrathoracic pressure, and sympathetic nervous system activity. These responses may contribute to the transient increase in arterial stiffness observed immediately following resistance exercise. Importantly, Jurik, Zebrowska & Stastny (2021) emphasize that these changes should not be interpreted as deleterious remodelling, but rather as short-lived hemodynamic adjustments to mechanical and pressor stress. Following the initial response, multiple modalities of exercise (including resistance exercise) are shown to induce post-exercise hypotension—a sustained reduction in resting blood pressure during recovery (Aly & Yeung, 2023). In resistance-specific contexts, Rezk et al. (2006) demonstrated that systolic blood pressure following heavier- and lighter-load resistance exercise may decrease or remain lower than a control condition for several hours. MacDonald (2002) outlined potential mechanisms underlying post-exercise hypotension, including persistent peripheral vasodilation (resulting from AMPK activation enhancing endothelial nitric oxide synthase activity (Chen et al., 2009), reduced sympathetic outflow, enhanced baroreflex sensitivity, and altered vascular responsiveness.

Thus, the acute vascular response to resistance exercise appears biphasic: an initial transient increase in arterial stiffness, followed by a reduction in resting blood pressure during recovery. Whether repeated exposure to these transient hemodynamic stresses contributes to longer-term vascular adaptation remains an important mechanistic consideration, but cannot be established from acute studies alone. The post-exercise hypotensive phase demonstrates an immediate haemodynamic effect that may be relevant to the longer-term cardiovascular benefits associated with regular resistance exercise (Jurik, Zebrowska & Stastny, 2021).

## Metabolic Responses

A single bout of resistance exercise elicits multiple acute metabolic responses that extend beyond localized muscular contraction. For example, muscle protein synthesis (MPS) is elevated following resistance exercise and represents one of the most extensively studied acute responses to mechanical loading (Phillips, 2014). Research suggests that MPS increases within approximately 3–6 hours (Phillips, 2014) and can remain elevated for up to 48 hours (Phillips et al., 1997; Atherton & Smith, 2012) following a single bout of resistance exercise. In contrast, nutrient driven MPS is of finite duration (∼1.5 h) despite surplus amino acid availability (Atherton & Smith, 2012). Mechanical tension, amino acid availability, and intracellular energy sensing converge on mTOR complex 1 (mTORC1), integrating anabolic signalling with nutrient status and cellular energy availability (Phillips, 2014; Bodine et al., 2001; Bond, 2016). While commonly discussed in the context of hypertrophy, the acute metabolic response to resistance exercise involves changes in protein turnover alongside broader alterations in substrate utilization and cellular energy sensing.

While MPS remains elevated following a bout of resistance exercise, glucose uptake also increases both with- and independent of- insulin to replenish muscular glycogen stores (Brown et al., 2020). Mechanistically, muscular contraction stimulates insulin-independent GLUT-4 translocation to the sarcolemma *via* AMPK activation and Ca2+-calmodulin–dependent pathways, enhancing glucose uptake independent of circulating insulin (Richter & Hargreaves, 2013). Brown et al. (2020) reported reductions in blood glucose concentrations for up to 24 h following a single resistance training session. Furthermore, glycogen depletion may further influence post-exercise insulin sensitivity and subsequent whole-body glucose disposal (Jensen et al., 2011).

Importantly, skeletal tissue participates in this metabolic recalibration through endocrine signalling. Acute mechanical loading has been shown to increase circulating osteocalcin, a hormone produced by osteoblasts that influences insulin secretion, insulin sensitivity, and energy expenditure (Lee et al., 2007; Scott et al., 2011). Osteocalcin enhances pancreatic *β*-cell proliferation and insulin production while improving peripheral insulin sensitivity in skeletal muscle and adipose tissue, thereby linking skeletal mechanotransduction to systemic metabolic regulation (Wei et al., 2014). The exercise-induced rise in osteocalcin may therefore represent an additional pathway through which skeletal loading influences post-exercise glucose regulation, alongside contraction-mediated GLUT-4 translocation (Mohammad Rahimi et al., 2021). These findings highlight a potential bone–muscle–pancreas signalling axis through which skeletal loading may influence acute metabolic regulation.

A further component of this energetic recalibration is excess post-exercise oxygen consumption (EPOC). Schuenke, Mikat & McBride (2002) demonstrated that metabolic rate could remain elevated for up to 48 h following high-intensity resistance exercise. This sustained increase in energy expenditure is thought to reflect, in part, ongoing restoration of phosphocreatine stores, lactate oxidation, catecholamine normalization, glycogen resynthesis, ion gradient restoration, and tissue repair processes (Børsheim & Bahr, 2003). Heightened protein turnover may also contribute to post-exercise oxygen consumption, as translational and proteolytic processes are energetically costly (Foureaux, Pinto & Dâmaso, 2006). Unlike steady-state aerobic exercise, resistance training induces intermittent high-force contractions that substantially perturb cellular homeostasis, necessitating prolonged recovery metabolism (Thomas et al., 2024).

Acute resistance exercise also influences appetite-regulating hormones and subjective satiety. Broom et al. (2009) and Balaguera-Cortes et al. (2011) reported modulation of ghrelin and peptide YY following resistance exercise, with evidence suggesting transient appetite suppression or neutral energy intake in the immediate post-exercise period. Exercise-induced changes in appetite signalling may interact with catecholamine responses and lactate dynamics to transiently suppress hunger perception (Schubert et al., 2014). Importantly, resistance exercise does not appear to provoke immediate compensatory appetite increases despite elevated energy expenditure (Panissa et al., 2024). Together these acute changes illustrate how resistance exercise can influence multiple components of post-exercise energy regulation, including energy expenditure, glucose metabolism, and appetite-related signalling.

## Exercise-Induced Hypoalgesia

Acute resistance exercise produces immediate and measurable reductions in pain sensitivity, a phenomenon known as exercise-induced hypoalgesia (EIH) (Wewege & Jones, 2021). Evidence across experimental studies suggests that a single bout of resistance training can increase pain thresholds, increase pain tolerance, and reduce perceived pain intensity in healthy individuals (Focht & Koltyn, 2009). While empirical research is limited, current findings are worthy of discussion. For example, in a controlled study examining resistance exercise performed at 75% of one-repetition maximum (1RM), pain thresholds significantly increased and pain ratings significantly decreased following exercise, regardless of whether the session occurred in the morning or evening (Focht & Koltyn, 2009). Dynamic circuit resistance exercise has also been shown to enhance pain tolerance. Baiamonte et al. (2017) reported significant increases in pain tolerance following a circuit resistance protocol, alongside elevations in heart rate, perceived exertion, and blood lactate. Notably, post-exercise lactate levels were positively correlated with pain tolerance, suggesting an association between metabolic stress and the hypoalgesia response. The evidence is broadly consistent with the findings of Wewege & Jones (2021), who conducted a systematic review of EIH and reported that dynamic resistance exercise produced small but significant reductions in experimentally induced pain in healthy individuals.

Mechanistically, EIH appears to involve complex neurophysiological pathways. Koltyn et al. (2014) demonstrated that increases in pain thresholds and reductions in pain ratings following exercise were accompanied by significant elevations in circulating endocannabinoids. Importantly, these hypoalgesia effects were not abolished by opioid receptor blockade, suggesting that non-opioid mechanisms, including endocannabinoid signalling, may contribute to acute exercise-induced hypoalgesia.

Collectively, the evidence suggests that a single session of resistance exercise can acutely reduce pain perception potentially through activation of endogenous pain modulatory systems. The potential practical implications of these acute changes for exercise adherence and rehabilitation remain to be established.

## Psychological Wellbeing

Acute resistance exercise can elicit rapid changes in psychological states, including reductions in anxiety and depressive symptoms and improvements in affect. Although much of the literature has focused on chronic training adaptations, a growing body of evidence suggests that even a single bout of resistance exercise can produce measurable acute changes in psychological outcomes.

With respect to anxiety, acute resistance exercise has been shown to produce reliable anxiolytic effects. In a focused review, Strickl & Smith (2014) concluded that single-bout resistance exercise reduces state anxiety across diverse populations and measurement tools. More recently, a randomized controlled trial in physically active adults demonstrated that a single session of resistance training significantly reduced anxiety symptoms within five minutes post-exercise (Amore et al., 2025). Although effects on depressive symptoms were more modest, reductions were still statistically significant supporting the role of acute resistance exercise in emotional regulation. Indeed, other articles have indicated that resistance exercise can acutely enhance positive affect and reduce tension (Cavarretta, Hall & Bixby, 2019). Beyond acute mood responses, resistance training has also been associated with improvements in self-perceptions. However, most evidence in this domain derives from repeated training interventions, with improvements in resistance training self-efficacy, physical self-worth, and global self-worth consistently documented (Collins et al., 2019).

Mechanistically, acute resistance exercise may reduce anxiety through modulation of hypothalamic–pituitary–adrenal (HPA) axis activity, alterations in cortisol dynamics, and shifts in autonomic balance favouring parasympathetic recovery (Amore et al., 2025; Athanasiou, Bogdanis & Mastorakos, 2023; Becker, Semmlinger & Rohleder, 2021). Resistance exercise can influence cortisol and other neuroendocrine responses that may be relevant to stress reactivity, although their specific contribution to post-exercise changes in anxiety remains unclear. Furthermore, acute reductions in somatic tension and physiological hyperarousal may decrease interoceptive threat perception, thereby lowering state anxiety.

In summary, the literature indicates that a single session of resistance exercise can acutely reduce anxiety, modestly improve depressive symptoms, and enhance affective states. These findings suggest that, in addition to its longer-term associations with mental health, a single bout of resistance exercise may produce immediate changes in anxiety and affective state.

## Cognitive Enhancement and Executive Function

Emerging evidence suggests that a single bout of resistance training can enhance executive function—particularly inhibitory control and cognitive flexibility—in healthy adults. A systematic review by Wilke et al. (2019) found that a single session of resistance exercise produced moderate improvements in global cognitive performance compared with a no-exercise control condition (standardized mean difference (SMD) = 0.56). Subdomain analyses revealed especially robust improvements in inhibitory control (SMD = 0.73) and cognitive flexibility (SMD = 0.36). Other narrative evidence is broadly consistent with these findings. Soga et al. (2018) reported that acute resistance exercise demonstrates the most consistent benefits for inhibitory control among the core executive functions. The authors propose that resistance exercise may influence attentional allocation, conflict monitoring, and prefrontal cortex engagement—processes central to executive regulation.

In a randomized crossover design, Engeroff, Banzer & Niederer (2022) demonstrated that acute resistance exercise significantly improved task-switching performance and attention. Similarly, Anders et al. (2021) examined the effects of high-intensity resistance exercise on multiple cognitive domains. Results indicated task-specific improvements, including enhanced mathematical processing throughput and faster reaction times post-exercise.

The physiological mechanisms underlying these acute cognitive changes remain incompletely understood. Transient changes in cerebral blood flow, catecholamine release, and neurotrophic factors have been proposed as potential contributors. Potential roles for brain-derived neurotrophic factor (BDNF), catecholamine availability, and insulin-like growth factor-1 (IGF-1) in exercise-related cognitive responses have also been proposed. However, evidence from populations with neurodegenerative disease and mild cognitive impairment (Romero Garavito et al., 2025; Liu et al., 2024) cannot be assumed to establish the mechanisms underlying acute cognitive responses to resistance exercise in healthy adults. Norepinephrine and dopamine have been implicated in executive control processes, while BDNF has established roles in synaptic plasticity and neuronal function (McMorris & Hale, 2015; Moore & Loprinzi, 2021). Whether acute resistance exercise-induced changes in these factors directly mediate improvements in executive function remains to be established.

Taken together, the available literature suggests that a single bout of resistance exercise can acutely enhance selected aspects of executive function in healthy adults. These improvements appear most consistent for inhibitory control and cognitive flexibility, with effects observable immediately post-exercise. Whether these acute changes have meaningful implications for academic, occupational, or other performance settings remains to be established.

## Sleep Architecture

Finally, while chronic resistance training has been associated with improvements in several sleep outcomes, including sleep onset latency, slow-wave sleep, and sleep disordered breathing, acute effects are less well studied (De Sá Souza et al., 2022; Kovacevic et al., 2018). However, there is a small body of research supporting acute effects of resistance exercise upon sleep architecture. Alley et al. (2015) reported that resistance exercise performed in the morning, afternoon, or evening resulted in fewer nocturnal awakenings compared to a non-exercise control day. Although sleep stage distribution (*e.g.*, REM and NREM proportions) was not significantly altered by timing, participants fell asleep faster, indicating improvements in sleep initiation. Furthermore, evening resistance exercise was associated with less wake time after sleep onset compared to control, suggesting enhanced sleep continuity (Alley et al., 2015).

Polysomnographic research examining evening resistance exercise provides important insight. In a controlled crossover study, Perrier et al. (2024) found that both evening resistance and endurance exercise altered sleep electroencephalogram (EEG) activity compared with a non-exercise control condition. While sleep efficiency was slightly reduced following both exercise modes, resistance exercise was associated with lower nocturnal cortisol levels than endurance exercise and resulted in lower beta activity (fast frequency brain waves associated with thinking, alertness, and focus) during N2 (stage 2 non-REM) sleep. Notably, N2 sleep accounts for 45–50% of total adult sleep and is critical for memory consolidation (Löwe et al., 2025). These findings suggest that resistance exercise performed in the evening can modify sleep-related EEG activity and may produce different nocturnal physiological responses from endurance exercise. Whether these differences translate into greater compatibility with sleep when exercise is performed close to bedtime requires further study.

Several mechanisms have been proposed through which exercise may influence subsequent sleep, including transient metabolic and thermal changes, and central nervous system fatigue (Driver & Taylor, 2000). Anti-inflammatory signalling, including potential roles for contraction induced myokines such as IL-10 and IL-1ra, has also been proposed as one pathway through which exercise may influence sleep regulation (De Sá Souza et al., 2022).

Overall, the limited available evidence suggests that a single bout of resistance exercise can influence subsequent sleep continuity and selected features of sleep-related EEG activity. Within the broader context of acute systemic responses, subsequent sleep may represent one potential pathway through which exercise-related physiological changes interact with recovery and next day function. However, the contribution of these acute sleep responses to longer-term metabolic, psychological, cognitive, or health outcomes remains to be established.

## Discussion

The evidence reviewed herein indicates that the physiological significance of resistance training extends beyond its well-characterized chronic adaptations. When considered collectively, the acute effects discussed in this review demonstrate that a single bout of resistance exercise can elicit responses across multiple physiological and psychological domains (Table 1; Fig. 1). These include myokine release (Zunner et al., 2022), transient vascular responses (Jurik, Zebrowska & Stastny, 2021), elevated post-exercise metabolic rate (Schuenke, Mikat & McBride, 2002), improved glycaemic control (Brown et al., 2020), stimulation of muscle protein synthesis (Phillips, 2014), reduced pain perception (Wewege & Jones, 2021), and changes in anxiety, mood (Strickl & Smith, 2014), cognitive performance (Wilke et al., 2019), and sleep related outcomes (Alley et al., 2015). However, these acute responses should not be interpreted uniformly as adaptive remodelling or beneficial effects. A single bout of resistance exercise imposes mechanical, metabolic, haemodynamic, and neuroendocrine stress, producing a combination of transient perturbations, compensatory responses, and immediate functional effects. The physiological significance of individual responses therefore differs across systems, and the extent to which specific acute responses contribute to subsequent chronic adaptations or health outcomes remains incompletely established.

The acute responses to resistance exercise may also have practical relevance, although this should be considered within the populations and outcomes studied. A single session appears to produce measurable changes in selected metabolic, physiological, and psychological outcomes without requiring repeated exposure over weeks or months. However, the clinical significance of these immediate responses, particularly in sedentary individuals and clinical populations, requires further investigation. Whether the rapid perception or experience of acute effects influences motivation, adherence, or subsequent exercise behaviour has also not been established. Resistance training should therefore not be viewed exclusively through the lens of long-term structural adaptation, as individual sessions can elicit measurable acute responses across multiple systems. When resistance exercise is repeated over time, these responses may represent potential contributors to longer-term adaptations and health benefits, although the specific causal pathways linking acute responses to chronic outcomes remain to be established.

Despite these promising findings, several limitations should be considered when interpreting the literature discussed in this review. Many acute resistance exercise studies have been conducted in relatively small samples, often involving young, healthy adults, which limits statistical precision and the extent to which findings can be generalised to older populations and clinical groups. Considerable heterogeneity in exercise protocols complicates cross-study comparisons and hinders the establishment of clear dose–response relationships. In addition, relatively limited acute data are available in clinical populations and individuals with cardiometabolic, musculoskeletal, or neurological conditions. Biomarker assessment windows are often restricted to the immediate or short-term post-exercise period, potentially overlooking sustained or delayed physiological effects. Finally, sex-based analyses remain underdeveloped, despite potential differences in endocrine responses and substrate metabolism.

At present, considerable heterogeneity in exercise protocols, including differences in exercise selection, volume, intensity, load, repetition duration, rest intervals, and timing of outcome assessment. This complicates cross-study comparisons and limits the ability to establish clear dose–response relationships. Future research could adopt more standardized and comprehensive designs incorporating 24–72-hour monitoring of metabolic, inflammatory, endocrine, vascular, and cognitive markers following a single resistance training bout. Longitudinal investigations examining the relationship between repeated acute responses and subsequent chronic adaptations will be important for determining whether, and to what extent, specific acute responses contribute causally to longer-term outcomes. Finally, dose–response studies are needed to determine how exercise selection, volume, intensity, load, and other protocol characteristics influence the magnitude and duration of acute responses. Such studies should also examine whether these responses differ according to age, sex, training status, and clinical condition, as the current literature is derived predominantly from relatively young, healthy populations.

## Conclusion

In summary, resistance training should not be conceptualized solely as a strategy for muscle hypertrophy or strength development. A single session can elicit measurable acute responses across multiple physiological and psychological domains, highlighting the broader systemic relevance of resistance exercise. These immediate effects provide a complementary perspective to the well-established chronic adaptations associated with regular participation. Although the extent to which repeated acute responses contribute to longer-term health outcomes remains to be established, this broader view reinforces the potential relevance of resistance exercise within preventive and rehabilitative medicine.

## Additional Information and Declarations

### Data Availability

The following information was supplied regarding data availability: This is a Literature Review.
